# The Medical Society

**Published:** 1899-10-14

**Authors:** 


### THE MEDICAL SOCIETY.

The opening meeting of the Medical Society of London was held last Monday, when a large number of members assembled to hear the presidential address given by Dr. Frederick Roberts, the popular new president of the society. Dr. Roberts dealt in turn with many of the most interesting medical topics of the day, and showed not only how great had been the changes during recent years in professional education and examinations, but also how great and solid an advance had taken place in medical knowledge and in our power over disease. He concluded his address with an admirably-worded eulogy of the present position of surgery, and of the enormous powers now possessed by the surgeon in relieving and often in actually curing disease.

On the conclusion of Dr. Roberts' address a discussion was opened on "Some Personal Experiences in Relation to Serum Therapeutics." Dr. Heron detailed his further experience in regard to the use of tuberculin (both old and new) in the treatment of lupus and pulmonary tuberculosis; after which Dr. Washbourn spoke of the sera of which he had had most experience. He especially pointed out the distinction which existed between the antitoxins of diphtheria and tetanus, which acted upon and neutralised the effects of the toxins produced by the microbes in question, and the anti-streptococcic and anti-pneumonic sera which interfered with the development of the microbes but left their toxins untouched. He went on to point out that useful as is the anti-diphtheritic serum, it is not, as some suppose, an altogether harmless agent. Indeed, in more than one case he thought its administration had had much to do with the subsequent death of the patient, so that, notwithstanding its proved efficacy, he was not prepared to say that it should be given in every case. He held out hope, however, that by deriving the serum from some other animal than the horse, which is at present employed in its preparation, we might in the future be able to obtain a more active form of antitoxin, one that will protect the nervous system better than the form at present in use. As to the other sera he would say that in certain cases the anti-streptococcic serum was most effective, but from the difficulty of being positive as to the exact form of streptococcus which was doing the mischief in a given case, it was not easy or, indeed, possible to say when such good results might be expected, and as regards the anti pneumonic serum he would withhold his judgment for the present.

